# Guy's Hospital Reports

**Published:** 1836-07

**Authors:** 


Art. VIII.—*Guy's Hospital Reports.* No. II. *April*, 1836. Edited by G. H. Barlow, m.a. Trin. Coll. Cam., Inceptor Candidate of the Royal College of Physicians; and J. P. Babington, m.a. Trin. Coll. Cam., Member of the Royal College of Surgeons. 8vo. pp. 225. *London.*

*St. Thomas's Hospital Reports.* By John F. South, Assistant Surgeon. No. II. and III. *January*, 1836. 8vo. pp. 117 and 116. *London.*

We have already stated the general objects and nature of these "Reports," which indeed their titles sufficiently indicate, and may now, without any formal prefatory remarks, give an abstract of the principal contents of the numbers before us. The minuter details we must necessarily avoid; the practical conclusions to be drawn from the various papers will direct the attention of our readers to the originals, and to these we principally limit our notice.

Mr. Bransby Cooper first relates three interesting cases of complicated fracture, which involve the very important question for surgical consideration, of when it may be right to attempt to save a limb; and when the limb is to be sacrificed to preserve life. In the first case, notwithstanding great laceration of the soft parts and other complicated injuries, the favourable constitution and temperament of the patient induced Mr. Cooper to attempt to save a limb which in a less happily disposed patient might have been unjustifiable. The patient left the hospital with every chance of completely regaining the use of her leg, and her general health was unimpaired. In the second case, immediate removal of the limb was demanded from the feeble powers of the patient. The third case was of an unusual character,—dislocation of the wrist with fracture of the radius. The ulnar artery too was torn through, an accident generally considered to indicate the necessity of amputation; but, observes Mr. Cooper, "it should be remembered that the free anastomosis of the vessels of the hand places this region of the upper extremity under the most favorable circumstances for reparation, and therefore the laceration of one of its principal trunks is not of the same importance as in other parts of the body: even the foot does not offer an analogous condition of circulation: for here the posterior tibial artery performs so much more important a duty than the anterior, that its division is infinitely more serious than when either the ulnar or radial arteries are lacerated, either of which are equally capable of sustaining the vitality of the hand."

Case 4.—*Wounded Knee Joint.*—This is an interesting case and one extremely creditable to Mr. Cooper. The patient was cured. The means employed were free bleeding, nauseating doses of antimony; the application of a splint to ensure perfect quietude of the limb, this being a very important feature in the management of such cases. Mr. Cooper believes that local irritants have not been sufficiently tried in such cases and advises large blisters. Tepid are preferred to cold lotions. Two other cases, with good engravings, are given by Mr. C. of retention of urine from fungus of the bladder, and enlargement of the prostate gland, and also a case of femoral hernia.

The next communication is from Dr. Hodgkin, whose talents and acquirements are well known to the profession. It is entitled "The history of an unusually formed placenta, and imperfect fœtus, and of similar examples of monstrous productions, with an account of the placenta and fœtus, by Sir Astley Cooper." Three engravings illustrate this paper. In reference to this case, Sir B. Brodie states in a letter to Sir Astley Cooper, that in 1809 he published an account of the dissection of a fœtus without a heart, and in which he believed that the circulation of the blood was carried on by the agency of the vessels only. Dr. Young afterwards suggested that the circulation in a fœtus of this kind is maintained by the heart of the twin fœtus with which such a monster is uniformly associated. Sir Benjamin was convinced by the argument of Dr. Young, and the dissection of Sir Astley Cooper proves the accuracy of his conclusions.

"The history of a compound fracture of the Patella, in a Letter from Mr. Lawrence, of Brighton, to Sir Astley Cooper," affords a gratifying proof of Mr. L.'s skilful management. In three months from the receipt of the injury, the patient was able to walk with a stick. "In looking over the report, the absence of swelling and inflammation, and the slight pain after so severe an injury, deserve notice. This may be attributed to the manner of applying the cold lotion. A trough containing the lotion was placed over the limb, from which hung several threads of worsted, acting as capillary syphons, and conducting as much of the lotion as was necessary to keep constantly wet a piece of thin linen laid upon the affected part: thus subduing the heat and inflammation by the uniform and constant evaporation produced." This plan of treating inflammations has been particularly advocated by MM. Josse, of Amiens, in a recent work, which will be noticed in our next number. It was introduced into the Brighton Hospital by Mr. E. Furner, the house surgeon. We have often seen a similar one employed at the Middlesex Hospital, and we cannot refrain from directing the attention of our readers to it, many of whom, like ourselves, must doubtless have had occasion to lament, that in both medical and surgical cases, the nurse or attendant generally makes the "cold" cloths a little warm with her hands, that they may suit her ideas of comfort for the patient.

The two next papers are by Mr. Key, and scarcely admit of condensation. The first is "Cases of accidents occurring to the large joints; and disease requiring removal by operation:" the second, "A case in which excision of the elbow joint was performed." They are very interesting, and sustain the high reputation of their distinguished author.

Mr. Blackburn's "Essay on the excision of diseased joints, read before the pupil's physical society," although highly creditable to its author, is hardly suited to a publication like the present.

Dr. Ashwell next offers some remarks "on the propriety of inducing premature labour in pregnancy complicated with tumour." Five cases are related, and are illustrated by four engravings. Dr. Ashwell's opinions upon this subject well deserve the attention of the accoucheur. The sixth case "Presentation of the arm, with small and deformed pelvis" is very interesting. In such instances, too, *the pelvic deformity having been ascertained* by the result of previous labours, Dr. A. thinks that "premature labour artificially induced, is the treatment for such contraction and deformity." The conjugate diameter of the pelvis was not quite two inches, ten lines. Dr. Ashwell's arguments in favour of the original practice he recommends are, to our minds, cogent. Accident led him to form his conclusions. In a case of tumour within the pelvis, he unintentionally separated the membranes, in making an examination, sufficiently extensively to induce premature labour—the best remedy, perhaps, for the complication. The recovery was good. A second case of this kind also occurred. Dr. A. says that to establish the propriety of inducing premature labour in such unfortunate and not very uncommon cases it is necessary to prove certain positions. That *when death occurs* after a labour so complicated the result *is only slightly, if at all referrible to the uterus,* which rarely sustains any serious injury, but is mainly produced by *inflammation, softening, and unhealthy suppuration* in the growth itself, and rapid sinking. That the pressure and contusion inflicted upon the morbid growth by the unyielding gravid uterus induces inflammation, suppuration, &c. which may be presented by the practice now first recommended, and which he believes deserves to be followed, for by it he may avoid the changes of a mischievous kind likely to be produced in the tumour during the last months of gestation, and certainly evading those greater dangers attendant upon parturition. Hitherto the management of such cases has been a subject of great doubt even with the most skilful and experienced practitioners.

The "Cases and Observations illustrative of Renal Disease, accompanied with the secretion of albuminous Urine, by Dr. Bright," are of much pathological value. Dr. B. states that that particular form of diseased structure of the kidney called "mottling, white degeneration," &c. is very common and very fatal. "I believe I speak within bounds, when I state that not less than five hundred die of it annually in London alone."

The second number of the Reports concludes with a "tabular view of the morbid appearances occurring in one hundred cases in connexion with albuminous urine, by Dr. Bright," and "Cases of Exostosis of the bones of the Face, disease of the Cranium, and fractures of the frontal and parietal bones requiring Operation, by Mr. Morgan."

The first paper of the second number of the St. Thomas's Hospital Reports we have already briefly noticed. In the second communication by Mr. South, of "Imperforate Anus successfully treated," we have an instance of that rare kind of malformation, in which no anus exists, but a small aperture in front of the scrotum through which fæculent matter passes. Mr. South performed an operation by which the most distressing consequences were removed.

The next paper is entitled "Simple Fracture of the Leg followed by Gangrene," by Mr. Green. Amputation performed by Mr. South. In the clinical observations by Mr. Green, the delicate surgical question is discussed, whether it is proper to amputate a limb when incipient gangrene is present. The patient died, but as far as we can judge, all was done that the limited power of man could do to secure a more fortunate result.

Dr. Roots's case of "Chronic Gastritis," offers nothing peculiar in itself, but affords Dr. R. the opportunity of making some apposite clinical remarks.

"Vomiting connected with Hysteria," reported by Mr. F. Le Gros Clarke. Creosote in one or two minim doses failed. After taking three minims the patient did not vomit for a long time. Dr. Roots in his remarks says that "the subsidence of the irritability of the stomach quite satisfied me that the complaint could not be of an inflammatory nature, because I never yet saw creosote taken into the stomach where was existing any thing like inflammation, but that it either produced an increase of the vomiting, or an increase of pain and heat in the stomach speedily after being taken."* A less experienced practitioner than Dr. Roots might easily have inferred the existence of inflammation from the tenderness on pressure at the epigastrium, abdomen and thorax. This symptom, however, was of an hysterical character, and depending merely upon that morbid sensibility of the sentient extremities of the superficial nerves which is so common in these cases, and so constantly mistaken and maltreated, to the destruction of the patient. If there is any practical subject more worthy the attention of a young physician than another, it is that of the wandering and often severe pains that attend "hysteria," and which, although temporarily relieved by bleeding, rarely require such means at all. Dr. Roots confesses that he has not the unlimited confidence in creosote, even in simple irritability of the stomach, that his colleague, Dr. Elliotson, reposes in it. He "has used creosote and failed; he has used it and succeeded." The doubts we have expressed, as to the never-failing virtues of creosote, in our notice of Dr. E.'s paper, are thus confirmed.

The cases of "Strangulated umbilical hernia," by Mr. Travers, offer no opportunity for comment. In a case of "strangulated inguinal hernia," there are several points worth attention. There was, first, resistance to the collapse and return of the intestine into the belly, after a dilatation free enough to admit the finger with perfect ease into the cavity. In cases of this kind, Blancard adopted the practice of puncturing in several places the protruded gut, to render it flaccid. By some, even in modern times, this practice has been followed, but Mr. Travers altogether disapproves of it. "It is dangerous and never can be called for in any possible emergency." The second point refers to the treatment after the operation, principally to the administration of purgatives and mercurials, which Mr. Travers condemns, and we are sure very justly, notwithstanding the high authority by which it is so unaccountably supported. Another excellent practical paper by Mr. Travers "On Perinæal Abscess, and Extravasation of Urine, arising from Stricture, Fistula, &c." concludes the number. The two following cases are very important.

"A lady had a recto-vaginal fistula, through which the feculent matter was continually passing. She was a delicate married person, with a young family, and suffered exceeding distress of mind, for reasons which may be readily conceived; and feeling that her person would become disgusting and her life valueless if this affliction continued, she consulted a surgeon of eminence, and implored him to devise some means of cure, stating her perfect willingness to endure whatever operation was necessary to that end. He reflected that the principle on which preternatural orifices or communications between canals are brought to heal, is that of rest; that we succeed in the cure of fistula in ano by suspending the habitual contraction and dilatation to which the parts are subject; and that this is followed by a healing process, if there be power in the system to effect it. He therefore suggested the free division of the sphincter ani, thinking it might possibly be followed by success. The patient most readily consented to the operation, and in the course of a few weeks was per-

* Dr. Roots's remark is confirmed by Dr. Elliotson. *Vide* our notice of his paper in the Med. Chir. Trans. p. 169.—Rev.

fectly cured, and remains so to this day. The other case, not less ingenious, was devised and accomplished by an old pupil of this hospital, who brought the patient to me for examination. There was a free communication between the bladder and vagina; of course on the anterior wall of the vagina. The large opening had been seared and stimulated, and the catheter worn; in short, all methods were tried in vain. What do you think this gentleman suggested? He asked her if she would submit to lie on her face for a couple of years. You will see at once what was the intention and effect of this posture. She consented, and in eighteen months was perfectly cured. An ugly puckering and knitting of the parts together, and some shortening of the passage has taken place during the healing; so that I think there would be great danger of a lesion of the cicatrix, if she were again to be with child; but the effect of continued rest in that position, to prevent the passage of urine through the opening, was to allow of such a cohesion of the parts that not a drop escaped, and the cure was established."—(P. 235.)

The third number of St. Thomas's Reports, we are compelled, by want of room, to pass over for the present. The following are the titles of the papers which it contains:—On Porrigo Lupinosa, and Hysterical Paralysis, by Dr. Roots. Compound Dislocation of the Clavicle, backwards, by Mr. Tyrrell. Chronic Dysentery, by Dr. Roots. Diseases of Joints, Mr. Tyrrell. Idiopathic Erysipelas treated with wine, Dr. Williams. Encysted Tumour—diffused Tuberculation, Mr. Travers.

Both of these publications maintain the high character they set out with, their contents being equally creditable to the authors and to the schools to which they belong. We must, however, repeat our wish that all our metropolitan hospitals would unite in the support of one more general and comprehensive publication.

